# Mechanical properties of plasma membrane vesicles correlate with lipid order, viscosity and cell density

**DOI:** 10.1038/s42003-019-0583-3

**Published:** 2019-09-13

**Authors:** Jan Steinkühler, Erdinc Sezgin, Iztok Urbančič, Christian Eggeling, Rumiana Dimova

**Affiliations:** 1 Theory and Bio-Systems, Max Planck Institute of Colloids and Interfaces, Science Park Golm, 14424 Potsdam, Germany; 2 MRC Human Immunology Unit, Weatherall Institute of Molecular Medicine, University of Oxford, Headley Way, Oxford, OX3 9DS UK; 3 Condensed Matter Physics Department, “Jožef Stefan” Institute, Ljubljana, Slovenia; 4 Institute of Applied Optics Friedrich‐Schiller‐University Jena, Max-Wien Platz 4, 07743 Jena, Germany; 5 Leibniz Institute of Photonic Technology e.V., Albert-Einstein-Straße 9, 07745 Jena, Germany

**Keywords:** Membrane biophysics, Membrane structure and assembly

## Abstract

Regulation of plasma membrane curvature and composition governs essential cellular processes. The material property of bending rigidity describes the energetic cost of membrane deformations and depends on the plasma membrane molecular composition. Because of compositional fluctuations and active processes, it is challenging to measure it in intact cells. Here, we study the plasma membrane using giant plasma membrane vesicles (GPMVs), which largely preserve the plasma membrane lipidome and proteome. We show that the bending rigidity of plasma membranes under varied conditions is correlated to readout from environment-sensitive dyes, which are indicative of membrane order and microviscosity. This correlation holds across different cell lines, upon cholesterol depletion or enrichment of the plasma membrane, and variations in cell density. Thus, polarity- and viscosity-sensitive probes represent a promising indicator of membrane mechanical properties. Additionally, our results allow for identifying synthetic membranes with a few well defined lipids as optimal plasma membrane mimetics.

## Abstract (web summary)

Steinkühler et al. use giant plasma membrane vesicles as a model system to study how plasma membranes respond to environmental changes, employing fluctuation spectroscopy and fluorescent probes. They show that the bending rigidity of plasma membranes vary with lipid order and microviscosity in correlated manner.

## Introduction

Plasma membrane (PM) remodelling governs essential cellular processes such as endocytosis and exocytosis, cellular division, nanoparticle and viral uptake and vesicle shedding^1–3^ and the energetic cost of elastic membrane deformation makes up a significant contribution to these processes^3^. However, progress in the field is limited by lack of methods to examine the PM mechanical properties^4^. Cellular complexity (in particular, the intertwinement of the PM with actin cytoskeleton and glycocalyx as well as its involvement in active processes such as endocytosis and exocytosis) poses a great challenge to measuring these properties in intact cells. To this end, model membrane systems such as giant unilamellar vesicles are applied. However, these synthetic systems lack the compositional complexity of the cellular PM. Here we show that isolation and study of PM blebs bridge the gap between mechanical manipulation of living cells and assays based on the use of synthetic cell-sized membranes, as exemplified by giant unilamellar vesicles^5–7^. After the initial reports^8^ about isolated PM blebs or giant PM vesicles (GPMVs), they have gained significant attention in the field of liquid–liquid phase separation in the membrane^9,10^. However, up to now they have rarely been used for the study of the PM mechanical properties^11^. An interesting aspect of GPMVs as a PM model is their compositional and structural complexity, closely representing the PM lipidome and proteome^12,13^, and the preserved lipid–protein^14^ and protein–protein^15^ interactions. As GPMVs are completely free of cytoskeletal support^16^, they can be used to distinguish the contribution of the PM from that of active processes or the cytoskeleton in mechanical manipulation assays (such as micropipette aspiration and tether-pulling) on living cells^17–19^.

In this way, GPMVs are ideally suited to address emerging questions of the role of lipids and lipid–protein assemblies in mechanical and structural cellular response. Here we use GPMVs to study the PM mechanics, membrane packing and viscosity while subjecting the cells to a range of different stimuli. By applying a spectrum of established characterization methods to GPMVs, we compare the readout from the used probes in PM and synthetic membranes and found a correlation of molecular order and viscosity with membrane mechanics across a broad range cell lines and culture conditions.

## Results

### Bending rigidity, lipid packing and viscosity measurements

**Fig. 1:**
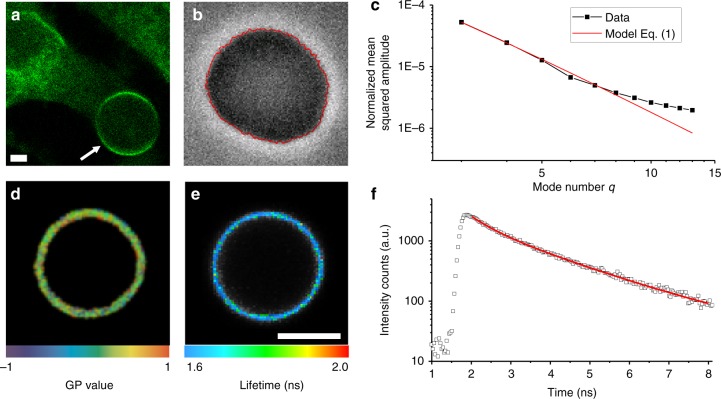
**a** GPMV formation. Chemically induced cytoskeletal cleavage of the plasma membrane results in the formation of vesicles (arrow), which subsequently detach from the cell (confocal cross-section). All experiments were conducted at room temperature where GPMVs exhibit one liquid phase as seen by homogenous Fast-DiIC18 (green) distribution, the variation of brightness along the contour is due to optical polarization effects. **b**, **c** Bending rigidity measurements by analysis of thermally induced membrane undulations. Detected membrane contour (red) is shown overlaid on the phase-contrast image in **b**. From the contour analysis, the power spectrum (black data in **c**) is obtained and fitted to Eq. 1 (red curve). **d** GP measurement on a GPMV membrane. Colour code corresponds to extracted GP map. **e**, **f** Fluorescent lifetime of molecular rotor embedded in the GPMV membrane. Colour code in **e** indicates average fitted rotor lifetime. Lifetime histogram from the whole membrane region of a GPMV (open points) and biexponential fit to the data (red curve) are shown in **f**. All scale bars indicate 5 μm

The bending energy of lipid bilayers are well described by the elastic sheet model with a single parameter, namely the bending rigidity *κ*, which takes values on the order of 20–30 *k*_B_*T* for single-component membranes made of typical phosphocholines^20^. Bending rigidities reported from tube-pulling measurements on cells appear to be a factor of 2–10 higher^19^, but the detected presence of actin and other cytoskeletal material in the pulled tubes is bound to influence the results. The bending rigidity of blebs bulging out from adhering cells was found strongly dependent on whether the bleb was expanding (softer and closer in bending rigidity to model lipid membranes) or retracting (stiffer)^21^. In the GPMVs studied here, the membrane (and the whole vesicle) is detached from the cytoskeleton. The GPMVs were isolated from adherent U2OS cells by chemically induced cleavage of the cytoskeleton from the PM^22^ (see “Methods” for details). When observed at room temperature using optical microscopy, U2OS GPMVs are mostly spherical and exhibit high optical phase contrast. Staining them with membrane dyes leads to homogenous dye distribution with no observable phase separation at room temperature (Fig. 1a, b). After slight osmotic deflation, membrane fluctuations are readily observed. Membrane fluctuations around the mean spherical shape were analysed (following the approach in ref. ^23^; see Fig. 1b, red line) and a power spectrum of the amplitude and energy of each fluctuation mode was obtained (Fig. 1c). The power spectrum deduced from the elastic sheet model (see Eq. 1 in “Methods”) was fitted with good agreement to the experimental data within the limiting factors of optical resolution and noise, which become apparent at higher mode numbers (high *q* values). Thus, we conclude that GPMV bending deformations up to length scales of the optical resolution (0.5 μm) can be described with a single parameter—the bending rigidity. It is worth noting that the bending rigidity is a material property of the membrane, while the membrane tension (which can be also assessed from fluctuation analysis) depends on the vesicle state and the forces acting on the membrane. In contrast to the more complex conditions in cells^24,25^, the membrane tension of GPMVs should be simply set by the area-to-volume ratio or, more broadly speaking, the shape of the vesicle. GPMVs used for fluctuation analysis were osmotically deflated and hence exhibited a low membrane tension of about 0.01 μN/m (which is similar in order of magnitude to that measured in cells^17^). As can be expected, the tension was not observed to vary with GPMV isolation conditions (Supplementary Fig. 1).

To correlate the continuum mechanical properties with molecular-scale descriptors, PM order and viscosity were also examined. Measurements were conducted at room temperature, above the temperature of phase separation of GPMVs^26^, to ensure homogeneous membrane properties across each vesicle. The lipid order was assessed from the spectral response of a polarity-sensitive membrane probe C-Laurdan, which exhibits a red-shifted emission in more disordered membranes^27,28^. For easier comparison, local spectral properties were converted to the so-called generalized polarization parameter (GP, Fig. 1d), which represents a relative index for molecular lipid packing. GP ranges between +1 and −1, with higher values representing higher lipid order^27,29^. To further interrogate the local PM viscosity, GPMVs were stained with a Bodipy-based molecular rotor^30,31^. The probe lifetime as explored with fluorescence lifetime imaging microscopy (FLIM) is sensitive to the membrane viscosity. Figure 1e shows an exemplary FLIM map, generated pixel-wise from respective histograms (Fig. 1f) of photon arrival times. This combination of methods was used in the next sections to obtain a detailed characterization of GPMVs isolated in varying conditions.

### Influence of GPMV isolation chemicals

**Fig. 2:**
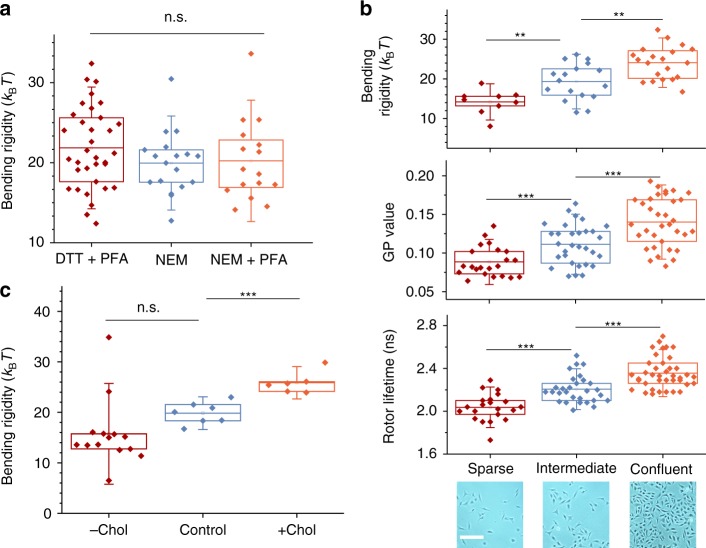
Effect of cell confluency, isolation chemicals and cholesterol content on the mechanical and molecular properties of PM membranes. **a** GPMV bending rigidity values for isolation using 2 mM DTT+25 mM PFA, 2 mM NEM and 2 mM NEM with subsequent addition of 25 mM PFA. **b** GPMV bending rigidity, GP and rotor lifetime for varying cell densities at the time of GPMV isolation by incubation with 2 mM DTT+25 mM PFA; phase-contrast images (scale bar: 200 µm) of adherent cells before isolation shown below. **c** Effect of cholesterol extraction (−Chol), enrichment (+Chol) or buffer only (control) treatment of cells before GPMV extraction. Each data point indicates one individual vesicle. Boxes have the conventional meaning of lower 25% and 75% quartile around the population mean value (middle line) and error bars indicate 1.5 std. dev

GPMVs can be prepared using various conditions^8^, but it is unclear how the different approaches affect the membrane mechanical properties. Modulation of elastic properties might be anticipated because changes in lipid phase by varying extraction methods are well documented. For example, GPMVs prepared with dithiothreitol (DTT) and paraformaldehyde (PFA) exhibit a demixing temperature about 10–20 °C higher than that of GPMVs derived using *N*-ethylmaleimide (NEM)^26^. DTT has been shown to affect lipid–lipid and lipid–protein interactions and to integrate directly into lipid membranes^32,33^. To figure out potential differences in mechanical properties, the bending rigidity of GPMVs formed using these two most widely employed methods was assessed. GPMVs prepared by incubation with DTT+PFA or NEM show only a small (<2 *k*_B_*T*) difference in bending rigidity (Fig. 2a). Apparently, the artefacts induced by DTT do not exhibit a strong effect on the bending rigidity.

We also questioned the effect of PFA^34^, which may be potentially inducing (membrane) protein crosslinking, even when used at much lower concentration than in cell-fixation protocols. GPMVs were extracted using NEM, and 25 mM PFA was added to the GPMV suspension followed by 1 h incubation. No significant influence of PFA on the bending rigidity was observed, indicating that potential effects of protein–protein crosslinking are small when considering the bending rigidity variance between individual GPMVs (Fig. 2a).

### Cell density modulates membrane properties

Adherent cells have been reported to adapt their membrane composition depending on the local cell density and time in culture after seeding^35–37^, but it remains unclear how PM elastic response is related to changes in membrane composition. We extended our studies to GPMVs isolated from cells at varying confluency but constant time in culture. We found a strong correlation between initial cell density and GPMV bending rigidity, with a trend for a stiffer phenotype at higher cell confluency (Fig. 2b). Interestingly, PM stiffening has been related to reduced cell migration^11^. The bending rigidity measured here for cells in sparse conditions are consistent with those reported for blebs expanding from adhering cells^21^. Note that significantly higher values for the bending rigidity of cell membranes have also been reported^19^, but these data were obtained from tube-pulling experiments where the detected presence of actin in the pulled tubes could explain the difference.

To further understand the relation between GPMV membrane physical properties and confluency, we assessed the PM order and viscosity, which were found to increase with cell confluency (Fig. 2b). These results are consistent with the finding that growth conditions modulate the phase transition temperature of GPMVs derived from cells at varied confluency^38^. We find that, in the GPMV model system, molecular membrane properties such as lipid order (reported by the C-Laurdan GP value) and dynamic parameter of microviscosity (assessed from lifetime of Bodipy rotor) correlate with the equilibrium elastic parameter of bending rigidity (Supplementary Fig. 2).

### Cholesterol content and temperature modulate PM bending rigidity

It is already well established that PM order (assessed by GP value of polarity-sensitive membrane probes, such as C-Laurdan) is related to cholesterol content in cell PMs and GPMVs^39,40^. Thus we questioned whether the bending rigidity of derived GPMVs is related in similar fashion to cellular PM cholesterol content. To this end, cholesterol concentration in the PM was directly altered by incubation of the cells with methyl-β-cyclodextrin before GPMV isolation; cyclodextrins are agents actively used for cholesterol exchange^41,42^. In the used conditions, about 60% cellular cholesterol was extracted from the PM^43^. GPMVs isolated from cholesterol-depleted cells (−Chol) appear softer while cholesterol enrichment (+Chol) stiffens GPMV membranes (Fig. 2c). It is tempting to speculate that the bending rigidities of cholesterol-depleted and -enriched GPMVs exemplify the respective bending rigidities of liquid ordered (Lo) (“lipid-raft like”) and liquid disordered (Ld) phases in the PM. For their ratio, we find *κ*_Lo_/*κ*_Ld_ ≈ 1.7. This stiffness mismatch is consistent with the membrane morphologies we observe in GPMVs exhibiting coexisting Lo–Ld phases (Supplementary Fig. 3): to minimize the bending energy, the lipid raft-like phase exhibits lower membrane curvature and curvature stresses preferably act on the softer non-raft phase bending it in the vicinity of the two-phase contact line, similarly to the behaviour observed in L_o_–L_d_ phase-separated synthetic model membranes^44,45^.

**Fig. 3:**
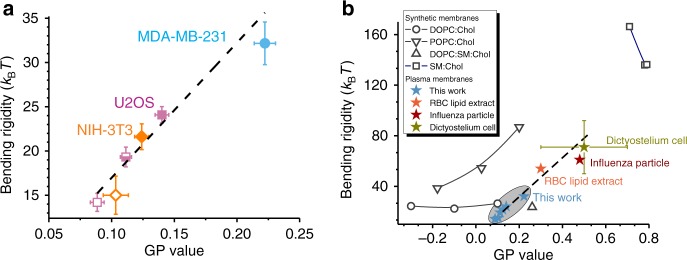
Correlation between plasma membrane lipid order, reported by C-Laurdan GP, and bending rigidity, and comparison to lipid-only membranes. **a** Correlation between mean values of C-Laurdan GP, bending rigidity obtained on GPMVs harvested from different cell lines (as indicated by the colour) and at varying cell density as indicated by the filling of the symbol: open—sparse, half-filled—intermediate, solid—confluent. The relation between GP values and the bending rigidity *κ* is well approximated by *κ*(GP) = (153 × GP + 2) *k*_B_*T* (adj. *R*^2^ ≈ 0.91) shown as dashed line. **b** Literature values for C-Laurdan GP and bending rigidity of DOPC:Chol (open circles) with increasing fraction of cholesterol from left to right 1:0, 7:3, 5:5 molar ratios, POPC:Chol (triangles) 1:0, 9:1, 2:1, DOPC:SM:Chol (solid square) 7:1:2 and SM:Chol (open blue squares) 9:1, 8:2, 7:3. Data from lipid vesicles (black symbols), RBC lipid extract (orange star), influenza particle (cherry) and Dictyostelium (ochre) are adapted from refs. ^20, 23^^,^^79–86^. Dictyostelium PM bending rigidity was estimated from talin-deficient cells^87^. Blue stars indicate values found for GPMVs at varying confluency in this study (same data as in **a**). Errors bars (std. error) are only shown if known or are significantly smaller than the size of the data point (**b**). The dashed line is identical to that in **a**

Apart from cholesterol content, membrane fluidity also changes with temperature. Generally, all experiments were conducted at room temperature (23 °C). However, when bending rigidity experiments were performed at physiological temperature, GPMVs were found to soften from *κ*(23 °C) ≈ (5.1 ± 1.2) × 10^–20^ J (data from Fig. 3a) to *κ*(37 °C) ≈ (2.3 ± 1.2) × 10^−20^ J (*n* = 6). This result is again consistent with reduction of membrane order (C-Laurdan GP value) at increased temperature^34,46^. Given these lines of evidence, we aimed to further explore the interdependence of lipid order (C-Laurdan GP value) and bending rigidity.

### Membrane order and bending rigidity correlate in a wide range of PM models

We set to explore how universal and quantitatively consistent the correlation between PM bending rigidity and molecular order (C-Laurdan GP value) observed in GPMV originating from U2OS cells is. To this end, NIH3T3 (fibroblast mouse embryo) and MDA-MD-231 (epithelial human metastatic) cells were cultured and GPMVs extracted by incubation with DTT/PFA (see “Methods” and Supplementary Fig. 4). Even though all cell models considered here were of different phenotype and morphology, their bending rigidity and C-Laurdan GP values are well fit by a single linear relation (Fig. 3a). Additionally, literature data for membranes composed of red blood cell lipid extracts, influenza virus particles (which bud from highly ordered PM domains) and in vitro Dictyostelium cells were considered (Fig. 3b). Extrapolation of the linear correlation observed for the three cell lines explored here fits the literature data for various PM models surprisingly well (Fig. 3b). The seemingly universal correlation calls for a rather general mechanism determining the relation between C-Laurdan GP values and bending rigidity in PM membranes.

In the commonly used elastic sheet model for bilayer bending, the bending rigidity scales as *κ* ~ *K*_*A*_*d*^2^ with chemical composition reflected in the material constant *K*_*A*_, which is the stretching elasticity constant, and bilayer thickness *d*, defined by the bilayer structure. Because of the quadratic dependence on *d*, it is tempting to assign a dominant role of the membrane thickness on the bending rigidity variations. Indeed, GP values also scale with membrane thickness, which is generally also a measure of membrane lipid order. However, what remains unclear is why and under which conditions the empirically defined GP value would show approximately the same *~d*^2^ scaling. An additional challenge is the chemical heterogeneity of lipids, which can be better understood by comparing our data to well-characterized synthetic lipid bilayers.

### Comparison to synthetic lipid membranes

Interestingly, in synthetic membranes, correlation between order, bending rigidity and cholesterol content was previously thought to be universal with cholesterol attributing stiffness and membrane thickening, see e.g. ref. ^47^. However, more recent studies revealed lipid-specific effects to membrane mechanics and molecular order^23,48,49^. For example, in synthetic lipid membranes made of POPC:Chol, lipid order (as measured by GP value) and bending rigidity are strongly positively correlated, while in DOPC:Chol and SM:Chol membranes they show no significant correlation (Fig. 3b); here POPC refers to palmitoyloleoylphosphatidylcholine, DOPC to dioleoylphatidylphocholine and SM to sphingomyelin. Similarly, membrane properties in more complex lipid compositions follow intuitive “universal” trends only along certain compositional trajectories^50^. Notably, bending rigidity and GP values of PM extracts studied here seem to follow a trend similar to that of POPC membranes with varying cholesterol fractions. It remains to be seen whether this reflects the abundance of PC lipids with single-chain unsaturation and cholesterol in the PM^13^. Note that absolute GP values and bending rigidities might vary depending on the exact measurement conditions, e.g. selection of the probe and spectral windows for GP calculation buffer conditions. For example, literature GP values for pure DOPC vary from −0.35^51^ and −0.38^52^ to −0.5^53^. Such deviations will skew the diagram shown and numerical values of the *κ*(GP) fit in Fig. 3b.

In absolute value, the GPMV data for bending rigidity and GP are closest to those of bilayers composed of DOPC:Chol at high (>30%) cholesterol fraction (Fig. 3b). However, it should be noted that a large fraction of GPMVs, mainly when isolated at sparse cell growth, exhibit a bending rigidity <20 *k*_B_*T*, which is lower than typical values of lipid membranes with a single *cis*-unsaturation in the acyl chains. Bending rigidities down to 10 *k*_B_*T* were measured for membranes made of lipids with polyunsaturated fatty acids^54^ and indeed polyunsaturated lipids are abundant in the PM and GPMVs^13^. Interestingly, also ternary mixtures of DOPC:SM:Chol fall into similar range of GPMV GP value and bending rigidity, while ternary mixtures with lower degree of lipid acyl chain unsaturation, e.g. SOPC:SM:Chol, appear to be stiffer^55^. Extraction of 60% PM cholesterol softened GPMV membranes by about 30%, which by absolute value is relatively little compared to the cholesterol dependence of POPC membranes but represents a considerably stronger stiffening effect due to cholesterol than for lipids with higher degree of unsaturation as shown for DOPC:Chol (Fig. 3b). (Poly)unsaturated lipids may act as a *buffer* to damp down variations in PM stiffness and in this way contribute to the homoeostasis of the cell. This might be one reason why cells invest energy into the synthesis and repair of these oxidation-prone lipids^56^.

Another contribution to the low values of bending rigidities of GPMVs might be the high membrane protein content (about 40% by mass is contained in GPMVs^57^), and proteins and peptides are often observed to reduce membrane bending rigidity^20,58–60^. GPMV membrane softening by transmembrane proteins is also consistent with lower bending rigidity values (*κ* ≈ 10 *k*_B_*T*) found in GPMVs derived from HEK cells overexpressing membrane protein^15^.

## Discussion

GPMVs, which retain most of the compositional complexity of native PM, seem to be compatible with the wide range of biophysical tools developed for synthetic giant unilamellar vesicles^5,7^. This makes GPMVs an interesting model system to study PM elasticity. We found that GPMV bending deformation can be described by a single parameter, the bending rigidity, the values of which are lower than for most of those obtained on synthetic lipid vesicles. This outcome should facilitate modelling and simulation studies of PM-like membranes and serve for comparison with synthetic lipid bilayers.

It is important to note that the inherent PM lipid asymmetry is not fully preserved in GPMVs (in particular the asymmetry resulting from flipping of phosphatidylserine lipids) and certain signalling lipids (such as PIP2) might be lost^61^. Even though studies on model membranes have attempted to characterise the effect of asymmetry on membrane phase state and mechanics^62–64^, the effects on bending rigidity resulting from partial loss of membrane asymmetry are difficult to predict. This is partly because bilayer bending rigidity cannot be considered as a simple superposition of the bending rigidities of the composing monolayers^65^. Previous studies have indicated large stiffening effects associated with membrane phospholipid asymmetry^66,67^, but these effects could be compensated by fast-flipping membrane components such as cholesterol^68^, which is abundant in GPMVs. Because we cannot yet control the extent of GPMV lipid asymmetry, this question is not directly addressed here.

Even though we have explored only three cell lines and can only speculate for universality of behaviour over any cell type, our results strongly suggest that external chemical and morphological stimuli, which have been previously reported to influence lipid order of cellular PM, also directly affect elasticity of PM-derived GPMVs, which points to a functional role of PM membrane stiffness or order. Potential roles of PM bending rigidity variation include cellular particle uptake or generation of exocytotic vesicles^2^ and regulation of receptor signalling via membrane fluctuations^15,69^. In the future, it would be interesting to investigate which signalling pathways modulate these membrane properties.

One important result of our work demonstrates that, in GPMVs, membrane structure (GP value, order) and viscosity, probed at molecular level by C-Laurdan and fluorescent rotors, are correlated to PM mechanical properties on a larger scale. This extends the use of C-Laurdan and molecular rotors as non-invasive tools to study elastic properties of PM in live cells, decoupled from the mechanical influence of cytoskeleton. It remains to be seen whether the correlation between GP value and bending rigidity holds also for organelle membranes, which are otherwise hard to probe directly. Further studies on the relationship between the compositional complexity and membrane mechanical properties will give invaluable information on the physiology of cellular membranes.

Finally, it should be noted, that the stable correlation between membrane order, dynamic microviscosity and equilibrium mechanical parameter of bending rigidity indicates a possible pathway for cells to sense and regulate membrane compositional variations via mechanosensing^70^.

## Methods

### GPMV isolation

Before experiments, U2OS, NIH 3T3 or MDA-MD-231 cells (obtained from ATTC) were plated under identical conditions in T-25 culture flaks and cultured in Dulbecco’s modified Eagle’s medium (DMEM) supplemented with 10% foetal bovine serum (Sigma F7524) and 1% Pen Strep (Thermo Fisher Scientific). For GPMV isolation at varying cell densities, cells were harvested from a confluent T-25 culture flask (about 2.7 × 10^6^ cells) and re-seeded in T-25 flasks after 1:10, 1:5 and 1:2 dilution in medium. After incubation for 24 h, GPMVs were isolated according to the protocol reported in ref. ^22^. U2OS cells were incubated with 2 mM DTT and 25 mM PFA at 37 °C for 1 h. Sometimes cells were labelled before GPMV extraction using the membrane dye Fast-DiIC18 (Thermo Fisher Scientific) by incubation of cells together with dye in phosphate-buffered saline buffer for 10 min at 4 °C.

### Fluctuation spectroscopy

GPMVs were extracted in nearly isoosmolar conditions (320 mOsmol/l, see buffer composition above). To allow for optically resolvable membrane fluctuations, the osmolality of the solution surrounding the GPMVs was slowly increased by evaporation. A 30-μl drop of GPMV suspension was left on a cover glass for 5 min before a chamber was formed by a top cover glass and “press-to-seal” silicon isolators (Sigma-Aldrich). Cover glasses were coated with bovine serum albumin (BSA) solution to suppress unspecific adhesion: cover glasses cleaned with ethanol were incubated for 30 min in 10 mg/ml BSA solution (fatty acid free, A1595 Sigma-Aldrich) and rinsed with double distilled water before usage.

The majority of defect-free GPMVs underwent optically resolvable fluctuations. Membrane bending rigidity was then measured by fluctuation analysis of the thermally induced motion of the membrane. Details of the method are published elsewhere^23^. Experiments were performed on an Axio Observer D1 microscope (Zeiss, Germany) using a ×40 objective in phase-contrast mode. Imaging was done using a low-noise liquid-cooled digital camera pco.edge 5.5. We acquired a total of 2000–4000 snapshots per vesicle with exposure time of 200 µs. Frame rates were varied between 20 and 100 frames per second with no difference in measured bending rigidity within the statistical uncertainty.

For a quasi-spherical vesicle of radius *R*_GPMV_, the dimensionless mean square amplitudes of the spherical harmonic modes behave as

1 $$\left\langle {\left| {u_{lm}} \right|^2} \right\rangle = \frac{{k_{\mathrm{B}}T}}{{\kappa \left( {l + 2} \right)\left( {l - 1} \right)\left[ {l\left( {l + 1} \right) + \bar \sigma } \right]}}$$

where $\bar \sigma = \sigma _{{\mathrm{eff}}}R_{{\mathrm{GPMV}}}^2/\kappa$ is the rescaled effective tension, which together with the bending rigidity *κ* is treated as a fit parameter.

To check for artefacts in the fluctuation spectra resulting from gravity-induced vesicle deformation, the criterion developed in ref. ^71^ was employed. The density of the GPMV cytosol-like interior was estimated by assuming a macromolecular volume fraction of 20 wt% with a density of 1.3 g/ml^72^ and a density of 1 g/ml of the outside buffer. The criterion by Henriksen et al.^71^ predicts negligible deformation for the typically small GPMVs with radii <10 μm and tension values estimated from the fit using Eq. 1.

### Modulation of cholesterol levels in cells

Cell-culture grade methyl-β-cyclodextrin (MβCD, Sigma) was either used as obtained from the manufacturer or pre-complexed with saturating concentration of cholesterol in sterile conditions^73^. Cells were washed and incubated with 10 mM of empty or cholesterol-loaded MβCD in DMEM buffer for 25 min at 37 °C. Control was incubated with DMEM buffer only.

### Measurements at elevated temperature

GPMVs were extracted as described above using DTT/PFA chemicals at 70% confluency. Sample temperature was controlled using a home-built chamber described in detail elsewhere^74^. Fluctuation analysis was performed as described above.

### GP and FLIM imaging

GPMV samples were split in half and labelled at room temperature with either C-Laurdan (0.4 µM) or molecular rotor (compound 1 in ref. ^30^, 0.05 µg/ml) for 30 min^22^. Although it is a relatively new probe, C-Laurdan is reported to respond to solvent polarity similarly to Laurdan^75–77^. Molecular rotors, on the other hand, change their lifetime depending on the viscosity of the environment^30,78^. For GPMVs labelled with C-Laurdan, spectral imaging was carried out with confocal microscope Zeiss 780 using ×40 water immersion objective (NA 1.2)^27^. C-Laurdan was excited at 405 nm and the emission was collected with a multichannel spectral detector in the range 410–540 nm, with a spectral step of 8.9 nm per channel. GP maps were calculated with the Fiji plugin freely available^27^. GP is calculated as follows: GP = (*I*_440_ − *I*_490_)/(*I*_440_ + *I*_490_) where *I*_440_ and *I*_490_ are the fluorescence intensities at 440 nm and 490 nm, respectively.

For FLIM imaging of the molecular rotor, labelled vesicles were transferred into glass-bottom eight-well microscopic slides (ibidi) and placed on an inverted laser-scanning confocal microscope (Leica SP8 STED) equipped with a time-correlated single photon counting module (PicoQuant) and a ×63 water immersion objective. Fluorescence in the equatorial plane of GPMVs was excited by a white light laser tuned to 488 nm and emission collected by a hybrid detector in the range 500–560 nm at a 160-nm pixel size. The photon streams were recorded until 300 photons per channel were detected. Average lifetime values for each GPMV were then extracted by fitting a bi-exponential decay curve to the histogram of data collected from pixels within the masked area along the perimeter of the GPMV, using the PicoQuant SymphoTime64 software. As noted previously^78^, the fast decay component of this probe in membranes (in our case with lifetimes around 0.6–0.8 ns and relative intensities around 20–30%) originates from a fraction of molecules that are insensitive to membrane viscosity due to their conformation (e.g. lying in the membrane plane). The values of fit component with longer lifetime were therefore used for further comparison.

### Statistics and reproducibility

Double-sided Student’s *p* test was used to estimate significance as indicated with n.s. *p* > 0.05, **p < 0.01 and ***p < 0.001 using OriginPro 2015. Experiments were generally conducted in biological triplicates, except those at varying growth density and cholesterol extraction, which were conducted in duplicates. Replicates were obtained from different passages of the same cell line. Sample sizes were not predetermined but all vesicles suitable for a measurement were considered in each sample within a 3-h window after the start of the experiment. Data collected on vesicles that did not fit to the analysis model was excluded (<10% of the sample size).

### Reporting summary

Further information on research design is available in the Nature Research Reporting Summary linked to this article.

## Supplementary information

Description of Additional Supplementary Files

Reporting Summary

Supplementary Data

Supplementary Information

## Data Availability

Source data for Figs. 1 and 2 and Supplementary Fig. 4 are provided as Supplementary data. All other data supporting the findings of this study are available from the corresponding author on reasonable request.

## Code availability

The custom analysis software used for bending rigidity measurements can be obtained from the corresponding author upon request.
