# Supplementary material for: Mechanical properties of plasma membrane vesicles correlate with lipid order, viscosity and cell density
Source: Commun Biol. 2019 Sep 13;2:337. doi: 10.1038/s42003-019-0583-3 (PMC6744421; doi:10.1038/s42003-019-0583-3)
Supplement: Supplementary file 1 — Description of Additional Supplementary Files [file 42003_2019_583_MOESM1_ESM.pdf]

Description of additional supplementary items

File type: Excel

Description: Supplementary Data

Source data for plots Fig 1: Example data for power spectrum of membrane fluctuations and FLIM rotor data including fit to the model

Source data for plots Fig 2: Rotor lifetimes (ns), GP values and bending rigidity (k_BT) for individual GPMVs in the conditions of sparse, intermediate and confluent cell density

Source data for plots Fig S4: Rotor lifetimes (ns), GP values and bending rigidity (k_BT) for individual GPMVs in the conditions of sparse, intermediate and confluent cell density
