# Supplementary material for: Mechanical properties of plasma membrane vesicles correlate with lipid order, viscosity and cell density
Source: Commun Biol. 2019 Sep 13;2:337. doi: 10.1038/s42003-019-0583-3 (PMC6744421; doi:10.1038/s42003-019-0583-3)
Supplement: Supplementary file 4 — Supplementary Information [file 42003_2019_583_MOESM4_ESM.pdf]

Supplementary Figures

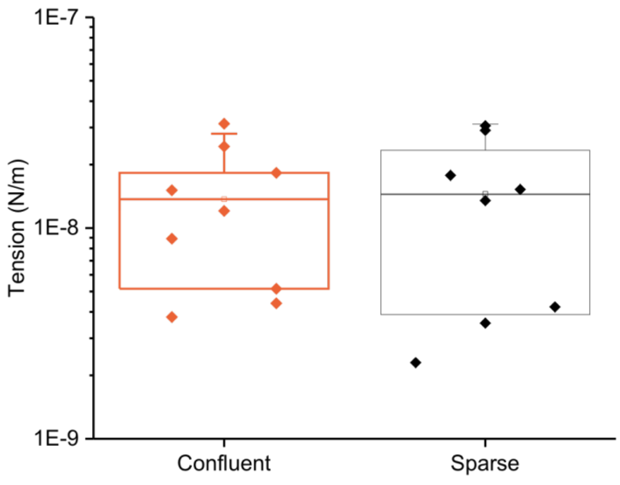

Supplementary Figure 1 – Typical membrane tension values measured by fluctuation spectroscopy. The two samples correspond to a subset of GPMVs from Fig. 2b and indicate the cell confluency at GPMV isolation (GPMVs were extracted by DTT/PFA). Each data point indicates one individual vesicle. Boxes have the conventional meaning of lower 25% and 75% quartile around the population mean value (middle line) and error bars indicate 1.5 std. dev.

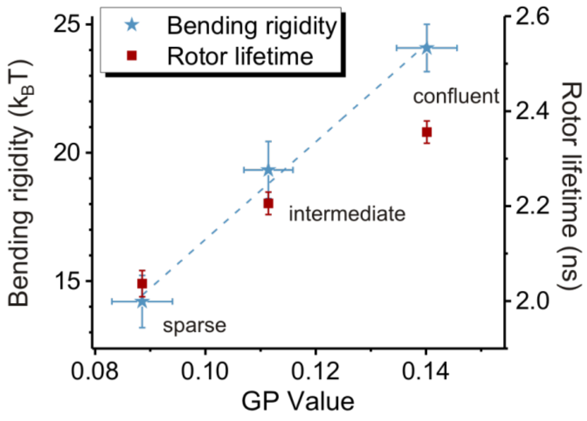

Supplementary Figure 2 – Correlation between values of bending rigidity, GP value and viscosity measured on U2OS cells at varying cell density. Error bars indicate std. error from mean (n=9,17,20 GPMVs).

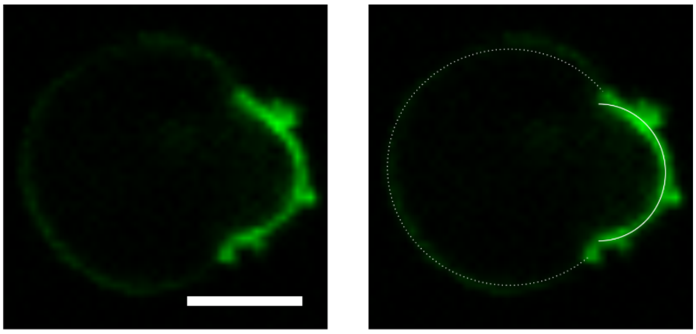

Supplementary Figure 3 – Confocal cross section obtained on a phase separated GPMV. At room temperature the majority of GPMVs exhibits one single fluid phase. However, a fraction of GPMVs was found to be phase-separated into two liquid phases. Fast-DiIC18 (dye) partitions into the liquid disordered phase (shown in green). In the right image the curvature of the two membrane segments is shown with solid and dotted contours. As it can be seen from their intersection, the liquid disordered phase (domain on the right side) deforms to match the curvature of the liquid ordered phase, indicating that it is energetically more favourable to bend the liquid discorded domain. Scale bar indicates 5µm.

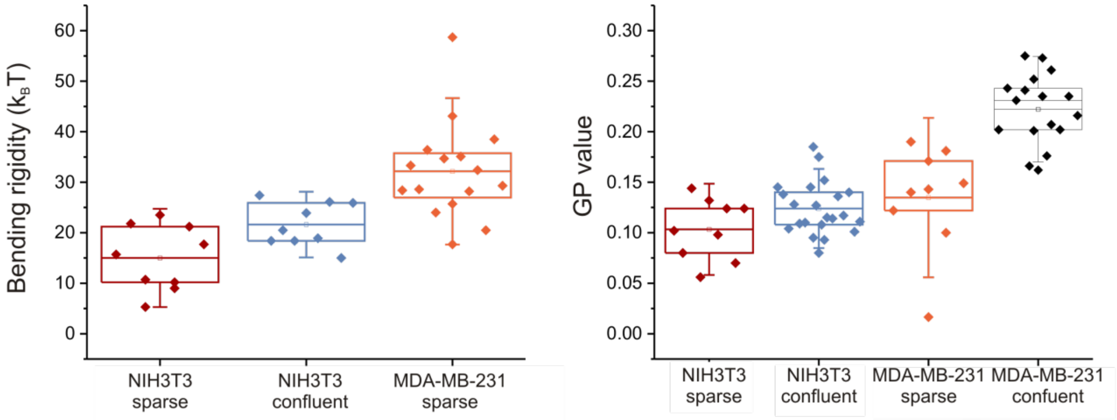

Supplementary Figure 4 - Effect of cell confluency on the mechanical and molecular properties of three cell lines. Each data point represents one measurement on a single GPMV. GPMVs were isolated using 2 mM DTT + 25 mM PFA. Boxes have the conventional meaning of lower 25% and 75% quartile around the population mean value (middle line) and error bars indicate 1.5 std. dev. Bending rigidity data on MDA-MD-231 cells at spare conditions could not be obtained due to low yield of suitable GPMVs.
